# Does Tension Headache Have a Central or Peripheral Origin? Current State of Affairs

**DOI:** 10.1007/s11916-023-01179-2

**Published:** 2023-10-27

**Authors:** Ángela Repiso-Guardeño, Noelia Moreno-Morales, María Teresa Labajos-Manzanares, María Carmen Rodríguez-Martínez, Juan Antonio Armenta-Peinado

**Affiliations:** 1 Department of Physiotherapy, Faculty of Health Sciences, University of Málaga, C/ Arquitecto Francisco Peñalosa, 3, 29071 Málaga, Spain; 2 Biomedical Research Institute of Malaga-Nanomedicine Platform (IBIMA-BIONAND Platform), 29590 Málaga, Spain

**Keywords:** Tension-type headache, Pathophysiology, Central dysfunction, Peripheral dysfunction, Nociception, Pain

## Abstract

**Purpose of Review:**

The aim of this narrative review is to analyze the evidence about a peripheral or central origin of a tension headache attack in order to provide a further clarification for an appropriate approach.

**Recent Findings:**

Tension headache is a complex and multifactorial pathology, in which both peripheral and central factors could play an important role in the initiation of an attack. Although the exact origin of a tension headache attack has not been conclusively established, correlations have been identified between certain structural parameters of the craniomandibular region and craniocervical muscle activity. Future research should focus on improving our understanding of the pathology with the ultimate goal of improving diagnosis.

**Summary:**

The pathogenesis of tension-type headache involves both central and peripheral mechanisms, being the perpetuation over time of the headache attacks what would favor the evolution of an episodic tension-type headache to a chronic tension-type headache. The unresolved question is what factors would be involved in the initial activation in a tension headache attack. The evidence that favors a peripheral origin of the tension headache attacks, that is, the initial events occur outside the brain barrier, which suggests the action of vascular and musculoskeletal factors at the beginning of a tension headache attack, factors that would favor the sensitization of the peripheral nervous system as a result of sustained sensory input.

## Introduction

Tension-type headache (TTH) is the most common headache along with migraine and its treatment continues to be a challenge for the healthcare community [1]. According to the Global Burden of Disease (GBD, 2019), headaches, including TTH, are one of the most prevalent conditions worldwide. The global prevalence of TTH is estimated at an average of 26.0% (22.7–29.5%), with a higher prevalence in women than in men [2]. The 2018 International Classification of Headache Disorders (ICHD) divides TC into three subtypes based on the frequency of episodes: infrequent episodic tension-type headache (IETTH), frequent episodic tension-type headache (FETTH), and chronic tension-type headache (CTTH) [3]. Although there are differences between the TTH subtypes, they all share the common feature of bilateral non-throbbing pain, of a crushing nature, with mild-to-moderate intensity and without worsening with physical activity or association with nausea or vomiting [3]. Mild nausea is only seen in the CTTH [3] subtype.

According to the 2018 ICHD-3 [3], CTTH appears as a result of the natural history of an episodic tension-type headache (ETTH) sustained over time, with a frequency of onset of 15 or more days per month for, at least, 3 months.

Comparing episodic tension-type headache (ETTH) and chronic tension-type headache (CTTH), it has been observed that headaches in patients with CTTH are more frequent, intense, and require more medical attention [4, 5]. Although there are few studies on the clinical characteristics of CTTH [4, 6, 7], they have been found to be similar to those of ETTH, except for greater pain intensity [6]. In a population sample, it has been found that pain in CTTH is bilateral in 88% of cases, of a pressure or hardening quality in 83%, and of mild or moderate intensity in 96% of cases [6]. In addition, in 71% of cases, it is not aggravated by routine physical activity, and in 58%, there are no accompanying symptoms such as nausea, photophobia, or phonophobia [6].

Among the comorbidities that have been associated with TTH are genetic comorbidities [8–15], bad habits [16], fibromyalgia [17], neck pain [18], low back pain [19, 20], and migraine [6].

Regarding genetic comorbidity, specific genes that can cause TTH have not yet been identified, but a possible contribution of the genotype of the 5-HTT gene-linked polymorphic region (5-HTTLPR) and the Val158Met COMT polymorphism (encoding of catechol-O-methyltransferase) in the risk of suffering from CTTH or its phenotype [13–15]. Furthermore, it has been suggested that the APOE-ε 4 gene could be protective against TTH [21].

Associated with bad habits, it has been seen that patient with TTH ingests more alcoholic beverages than patients with chronic migraine (CM) [16].

Fibromyalgia has also been suggested as an important comorbidity of TTH, with a prevalence two times higher in patients with CTTH compared to those with ETTH, which could be explained by a shared mechanism of central sensitization [17].

On the other hand, some investigations speak of the comorbidity of TTH with neck pain and low back pain, since almost 90% of people with TTH have comorbid neck pain [18], and approximately 80% of people with TTH have low back pain [20].

Concerning migraine, a population study found that 83% of individuals with migraine in the last year also experienced tension-type headache (TTH) [6].

Increased sensitivity to pressure of the pericranial musculature has been observed in both ETTH and CTTH patients, not only during a CT attack but also on a regular basis [8, 22–29].However, compared to ETTH patients, CTTH patients have greater sensitivity to pressure of this muscle [23, 29, 30]. The literature suggests that pressure sensitivity increases proportionally to the severity and frequency of TTH episodes [9, 29].

Some studies indicated that the increased sensitivity to pressure of the pericranial musculature in patients with CTTH is a consequence of the progression from ETTH to CTTH and not the cause [23, 26, 29–34].

Despite the fact that TTH constitutes one of the most common headaches, the debate as to whether TTH attacks are initiated by peripheral or central factors remains unresolved [33, 34].

## Search Strategy and Selection Criteria

The search was carried out in MEDLINE and ProQuest (from databases inception to March 30, 2023) for original research articles, systematic reviews, and meta-analyses. The search term “Tension-type headache” was used in combination with the terms “Epidemiology,” “Pathophysiology,” “Risk factors,” “Burden,” “Central dysfunction,” “Peripheral dysfunction,” “Nociception,” and/or or “Pain.” Those studies that dealt with the differences between the central and peripheral origin of headaches were selected. We preferably selected publications from the last 10 years, but did not exclude commonly referenced and highly regarded older publications. Reference lists of articles identified by this search strategy were also searched, and those found to be relevant were selected considered relevant.

## Do TTH Attacks Have a Central Origin?

### Central Mechanisms

In TTH, it is believed that the pain experienced during the attacks is related to the malfunction of some supraspinal structures [34–38]. In patients with TTH, it has been found that the pain inhibitory mechanism called diffuse nociceptive inhibitory control (DNIC) does not work properly [20, 23, 29, 36, 39–41].

The DNIC is a mechanism of the central nervous system that helps to reduce the perception of pain. In TTH patients, a decreased ability of the supraspinal structures to perform this task effectively has been observed, which may contribute to the onset and persistence of tension headache attacks [32, 42]. However, the explanation of the factors underlying the dysfunction of the DNIC is still being studied [43].

Henceforth, morphologically, a reduction in the density of the gray matter of the primary somatosensory cortex has been demonstrated, as well as an increase in the density of the gray matter of the bilateral anterior cingulate cortex and the anterior insula in patients with HHT versus controls. These morphological changes have only been recorded during TTH attacks, being absent when the TTH episode did not occur [44]. This data supports the theory that the cingulate cortex and the insula, both areas recognized for contributing to the cognitive and affective processing of sensory information, play a fundamental role in the initiation of a tension headache attack [44–46]. However, the factors that trigger a TTH attack have not yet been elucidated [33, 34].

Endogenous opioids and neuropeptides such as calcitonin gene–related peptide (CGRP), substance P, neuropeptide “Y” (NPY), and vasoactive intestinal peptide (VIP) have important roles in modulating nociceptive signaling and have been investigated in relation to the pathophysiology of headaches, including TTH. Although some studies have found altered levels of these neuropeptides in patients with TTH [47–50], others have found normal levels suggesting that its role in TTH is still unclear [51–57].

Serotonin is another neurotransmitter that has been investigated in connection with TTH. Studies have yielded conflicting results, but ETTH patients have been found to have increased platelet and plasma serotonin levels, as well as decreased platelet serotonin uptake compared with controls [58, 59]. Serotonin levels in the cerebrospinal fluid (CSF) of TTH patients have been shown to be significantly lower than in healthy individuals, suggesting that serotonin deficiency may play an important role in the pathogenesis of TTH [37]. In addition, some drugs that increase serotonin levels, such as selective serotonin reuptake inhibitors (SSRIs), have been shown to be effective in the treatment of TTH [37]. Serotonin also influences regulating mood, sleep, anxiety, appetite, and pain perception. In relation to TTH, it has been proposed that the decrease in serotonin levels in certain areas of the brain of these patients could be associated with psychosomatic factors and sleep disorders, factors that could favor the central origin of a TTH attack [34, 37].

### Core Risk Factors

Evidence favoring a central origin of a tension headache attack includes both psychosomatic factors and sleep disturbances that would favor activation of structures within the central nervous system at the onset of and during a tension headache attack [34].

### Psychosomatic Factors

Imaging studies in patients with TTH have suggested a fundamental role for the anterior cingulate cortex and the insula, both areas recognized for contributing to the cognitive and affective processing of sensory information [45, 46]. In this regard, stress remains one of the most commonly recognized precipitating psychosomatic factors in the onset of a TTH attack [60–62]. Furthermore, it has been shown that cognitive stress can increase muscle pain in TTH patients compared to controls [63]. Physiologically, stress can trigger or aggravate headache by increasing muscle contraction, releasing catecholamines and cortisol, peripherally sensitizing, and/or affecting central pain processing [64]. The long-term release of corticosteroids in those patients with chronic stress could cause tissue damage and an increase in pain perception [65, 66]. In TTH, stress has been shown to trigger higher rates of pericranial muscle pain compared to controls [63]. However, and contrary to this theory, there are authors who have not found a relationship between stress and the alteration of the diffuse nociceptive inhibitory control [67], so the pathophysiological mechanism by which stress can act as a trigger for TTH and its role with respect to the limbic system and supraspinal mechanisms has not yet been elucidated [33]. Likewise, there is no evidence to support a relationship between stress and increased pericranial EMG activity in patients with TTH, so its role in the pathophysiology of TTH remains uncertain [63, 68]. Therefore, the pathophysiological mechanism by which stress can act as a trigger for TTH and its role with respect to the limbic system and supraspinal mechanisms have not yet been elucidated [33]. Likewise, there is no evidence to support a relationship between stress and increased pericranial EMG activity in patients with TTH, so its role in the pathophysiology of TTH remains uncertain [63, 68]. Therefore, the pathophysiological mechanism by which stress can act as a trigger for TTH and its role with respect to the limbic system and supraspinal mechanisms have not yet been elucidated [33]. Likewise, there is no evidence to support a relationship between stress and increased pericranial EMG activity in patients with TTH, so its role in the pathophysiology of TTH remains uncertain [63, 68].

On the other hand, it has been seen that psychosomatic factors such as depression can increase sensitivity to the perception of pain [34]. Population studies have shown that both depression and anxiety are more prevalent in individuals with TTH than in the general population without headache [69–73]. Cross-sectional research suggests that depression and anxiety are also associated with the frequency and severity of TTH attacks [74]. However, it is not possible to determine a causal relationship between psychosomatic factors and TTH based on cross-sectional data [34]. A longitudinal study showed possible bidirectional effects between psychosomatic factors and the characteristic pain of patients with TTH [75].

In this context, the correlation between depression/anxiety disorders and primary headache disorders, such as tension-type headache and migraine, has been extensively documented in clinical settings. Although psychiatric disorders rarely serve as the sole cause of headache symptoms, their comorbidity can significantly influence patient outcomes and treatment strategies [76]. Particularly, individuals with primary headache disorders and concurrent multiple psychiatric disorders require thorough evaluation and proper management, especially in cases involving medication misuse or insufficient response to conventional headache treatments [77].

In another longitudinal study conducted in patients with TTH, it was suggested that emotional factors play an important role in the perceived pain experience. Along the same lines, high levels of depression and neuroticism have been associated with increased sensitivity to pain in the cephalic and extracephalic regions both in individuals with TTH and in individuals with migraine [69], which would corroborate that the way in which the fact that pain is faced or managed affects the perception and modulation of pain in patients with TTH [75].

### Sleep Disorders

Recent population studies have revealed bidirectional comorbidity between sleep-related factors and TTH, suggesting physiological mechanisms of shared pathways [69–73]. This association could be explained by the participation of common neurotransmitters and brain networks, including the hypothalamus and the brainstem, in the pathophysiology of both diseases [78, 79].

Sleep apnea, insomnia, insufficient sleep, poor sleep quality, restless legs syndrome (RLS), and excessive daytime sleepiness (EDS) have been associated with the initiation and exacerbation of TTH attacks [79]. Likewise, it has been suggested that sleep disorders can lead to an alteration in the functioning of the supraspinal structures related to the perception and modulation of pain in CTTH [80, 81]. In this framework, it has been suggested that adequate treatment of sleep disorders and regulation of sleep/wake cycles can improve the management of TTH [79]. However, the role of sleep disorders in the progression from episodic tension-type headache to chronic tension-type headache is still a matter of debate [34].

### Central Management and Pharmacological Treatment

Tricyclic antidepressants, such as amitriptyline and mirtazapine, have been substantiated as effective therapies for tension-type headache (TTH) due to their demonstrated benefits in reducing both the frequency and intensity of symptoms [34]. Despite potential side effects, including drowsiness, weight gain, and sedation, mirtazapine presents better tolerability compared to amitriptyline, rendering it a recommended option, particularly for specific patient groups, such as the elderly [34]. Conversely, muscle relaxants have shown limited efficacy in alleviating TTH symptoms [34].

### Do TTH Attacks Have a Peripheral Origin? Peripheral Mechanisms

In tension headache, nociception of the craniocervical musculature is transmitted peripherally through thin myelinated A-delta fibers and unmyelinated C fibers [33]. In normal situations, thick myelinated fibers, such as the A-alpha and A-beta mechanosensitive fibers, only carry innocuous stimuli [33]. However, the literature suggests that in cases of sustained sensory input from the musculature, abnormal nociception occurs not only from A-delta and C fibers, but also from low-threshold A-beta mechanosensitive fibers [33, 82]. According to this concept, the increased sensitivity to pressure of the pericranial musculature in patients with CTC could be due to sensitization of the peripheral nervous system as a result of sustained sensory input from the involved musculature.

### Peripheral Risk Factors

The evidence that favors a peripheral origin of tension headache attacks, that is, the initial events occur outside the brain barrier, includes both the action of musculoskeletal factors and the action of vascular factors, the latter to a lesser extent [34]. In both cases, the theory of a peripheral origin would support the sensitization of the sensory afferents of the trigeminal-cervical nucleus at the onset of a tension headache attack [33, 34].

### Vascular Factors

It has been suggested that hemodynamic changes in the vascular supply to the brain may activate trigeminal nociceptors and cause headache [84]. In this sense, it has been observed that patients with ETTH present a small increase in the blood flow velocities towards the anterior, middle, and posterior cerebral arteries, while patients with CTTH present small alterations in the blood flow velocities of the basilar and posterior cerebral arteries. The mean brain was compared with controls [85–87]. However, it seems that the cerebral vascular input is more important in the initiation of migraine attacks than in TTH [88].

Some authors suggest that the vascular changes observed in patients with TTH could be due to alterations in the activity of CGRP [89]. Contrary to this theory, several studies have consistently shown that patients with ETTH and CTTH present normal CGRP levels during TTH attacks, both in blood and in cerebrospinal fluid [50, 52, 53]. However, CGRP has been identified as an important migraine mediator, since its release has been shown to correlate with the onset of migraine attacks and its inhibition can reduce the frequency and intensity of their episodes [7, 90].

### Musculoskeletal Factors

Myofascial trigger points (MTrPs) may be important in the pathophysiology of TTH [34]. Activation of the MTrPs of the craniocervical musculature has been observed to reproduce the characteristic pain patterns of tension headaches [12, 91–96]. Along these lines, a correlation has been suggested between the number of active MTrPs in the craniocervical musculature and the degree of pericranial sensitivity presented by patients with TTH [91, 94, 96–102]. However, although the action of the active MTrPs of the craniocervical musculature seems to be closely related to the initiation of THT attacks [96], it is not clear that the isolated summation of these MTrPs can serve as a marker of the transition from ETTH to a CTTH as some authors have suggested [97–99, 103, 104] since researchers have shown that there is no significant relationship between the number of MTrPs and the frequency of TTH episodes [39]. Therefore, the most accepted theory to date focuses on the fact that it would be the perpetuating action of the active MTrPs rather than their spatial summation, which would give rise to the evolution of an ETTH to a CTTH [37, 91–95, 97, 101, 103, 105–107]. Taking the latter into account, the debate on which musculoskeletal factors could be involved in the initiation of a TTH attack is still open [36, 108–112].

The literature suggests that among the factors that could trigger the activation of active MTrPs are cumulative trauma and sustained postures [113]. In addition, both cumulative trauma and sustained postures are responsible for the appearance of “satellite” or “latent” MTrPs, consequently generating greater spatial summation of muscle MTrPs [114] and therefore greater pericranial sensitivity[91, 93, 94, 96, 98–102].

On the other hand, it is known that, compared to healthy subjects, patients with TTH present a more forward position of the head and decreased cervical mobility [115]. In this sense, some authors have suggested a correlation between structural alterations of the craniomandibular region with changes in head posture and motor control of the craniocervical muscles [95, 115–117]. In this line, several investigations have correlated the activity of the craniocervical musculature with structural parameters of the craniomandibular region, such as the length of the body of the mandible or the gonial angle [118, 119]. Likewise, the position of the mandible at rest also seems to induce changes in the posture of the head and upper thoracic region [118, 119].

### Peripheral Management and Non-pharmacological Treatment

Aerobic exercise and strength training have shown promising potential in reducing the frequency and impact of tension-type headaches (TTH). However, there is currently no standardized physical therapy protocol specifically tailored for tension headaches. Nevertheless, various techniques studied to date have targeted the cranio-cervical-mandibular region using diverse approaches [120]. In particular, the management of cranio-cervical-mandibular myofascial trigger points has demonstrated significant effects in terms of reducing pain intensity and decreasing the frequency of headache episodes in the short and medium terms [34, 120].

In the context of physical therapy, it has been observed that techniques focusing on addressing the cranio-cervico-mandibular region offer benefits in terms of reducing the intensity, frequency, and duration of tension headache episodes [34, 120]. Additionally, aerobic exercise and strength training have shown promise in mitigating the frequency and impact of TTH on the daily life of affected patients [34].

## Lessons Learned and Future Directions

### TTH Attacks Have a Central Origin

The pain experienced by TTH patients is believed to be related to malfunctions of supraspinal structures, including diffuse nociceptive inhibitory control (DNIC). In this context, morphological changes of the gray matter have been found in areas recognized for contributing to the cognitive and affective processing of sensory information [44–46], changes that only occurred during TTH attacks [44].

On the other hand, it has been observed that patients with episodic tension-type headache (ETTH) have low levels of serotonin [58, 59], levels that are related to psychosomatic factors such as stress, depression or anxiety, and sleep disorders [37]. In this sense, a recent study has shown significant associations between the appearance and exacerbation of tension headaches and different sleep disorders [79]. Likewise, it has been suggested that sleep disorders can lead to an alteration in the functioning of the structures supraspinal nerves related to pain perception and modulation in TTH [80, 81]. However, the involvement of sleep disorders in the evolution from ETTH to CTTH is still a matter of debate.

On the other hand, it has been thought that factors such as stress could be a precipitating factor in an initial attack of tension headache [60–62] since the release of catecholamines and cortisol that this generates could affect the central processing of pain [64–66]. However, and contrary to this belief, there are authors who have not found a significant association between stress and the alteration of the diffuse nociceptive inhibitory control [67], so the pathophysiological mechanism by which stress can act as a trigger for TTH and its role with respect to the limbic system and supraspinal mechanisms has not yet been elucidated [33, 63, 68].

Following this line of thought, population studies have shown that people with TTH have a higher incidence of anxiety and depression than those without headache [69–73]. In this sense, there has been talk of a possible association between the frequency and severity of TTH attacks and the presence of anxiety and depression [75]. However, this theory is ambiguous since there are authors who have suggested that these associations could be influenced by the coexistence of central dysfunctions associated with migraine, which is highly comorbid with depression and anxiety [77, 78].

In line with this line of research, it has been suggested that emotional factors play an important role in the experience of pain perceived by patients with TTH, that is, the emotional state regulates the way in which pain is faced or managed and it can affect the perception and modulation of TTH [75]. However, whether a tension headache attack is initiated by emotional factors is uncertain.

### TTH Attacks Have a Peripheral Origin

The pain experienced by TTH patients is thought to be related to peripheral sensitization due to sustained sensory input from the craniocervical musculature, both from high-threshold mechanosensitive neurons and low-threshold mechanosensitive neurons. The perpetuation over time could be responsible for increased pericranial sensitivity in patients with CTTH [22, 24, 33, 83].

Musculoskeletal factors, especially active MTrPs of the craniocervical musculature, seem to play an important role in the pathophysiology of TTH, since the referred pain they generate is similar to the characteristic pain pattern of TTH [12, 91–96]. In this sense, and despite the fact that a correlation has been observed between the number of active MTrPs in the craniocervical musculature and the pericranial sensitivity present in patients with TTH [91, 93, 94, 96, 98–102], the spatial summation of MTrPs does not seem to be related to the frequency of TTH attacks [39]. Accordingly, it has been suggested that the frequency of tension-type headache attacks, and thus the transition from episodic tension-type headache to chronic tension-type headache, would be more correlated with those factors that trigger the activation of the MTrPs than with the isolated presence of MTrPs in the musculature [37, 91–95, 97, 101, 103, 105–107]. In this sense, it has been suggested that both cumulative trauma and sustained postures could be factors responsible for the activation of MTrPs [113]. Correspondingly, it has been seen that both cumulative trauma and sustained postures generate “latent” trigger points, consequently causing greater spatial summation of MTrPs [114]. In this line, it has been seen that structural alterations in the craniomandibular region can affect both the normal posture of the head and the motor control of the craniocervical musculature [95, 115–117]. Following this thought, there are investigations that show a correlation between craniofacial morphology and the activity of the craniocervical musculature [118, 119]. In the same way, the position that the mandible adopts at rest also seems to produce changes in the posture of the head and upper thoracic region [118, 119].

On the other hand, vascular hemodynamics as a possible risk factor in the onset of a tension headache attack has been the subject of debate. In this sense, it has been suggested that an increase in blood flow to the cerebral arteries could activate the trigeminal nociceptors and cause headache [85–87] although this theory seems to be more important in the onset of migraine attacks than in the TTH [88].

## Conclusion

Considering the literature on this topic, it is likely that both peripheral and central mechanisms are involved in a tension headache attack, e.g., peripheral nociceptive input is required for pain transmission and cortical activity is required for pain perception. However, the debate as to whether tension headache attacks are initiated by peripheral or central factors remains unresolved. In fact, some authors speak of the action of peripheral factors such as the postures maintained and muscular tension, at the beginning of a TTH attack, while other authors suggest that central factors, such as emotional stress, depression, or disorders of sleep, may play an important role in the initiation of a TTH episode.

Although it has been observed that certain structural parameters of the craniomandibular region seem to correlate with head posture and with the activity of the craniocervical musculature, it has not yet been carried out enough studies that compare these structures in TTH patients and controls. The cephalometric study of a human model with CTTH could clarify whether craniomandibular structural factors could favor tension-type headache.

In conclusion, TTH is a complex pathology with a probably multifactorial origin in which both peripheral and central factors may be involved. Therefore, it is important to continue research to improve the understanding of this pathology and to be able to develop better treatments and preventive strategies.

## Data Availability

No data was generated for this manuscript.
